# Disruption of the endothelin A receptor in the nephron causes mild fluid volume expansion

**DOI:** 10.1186/1471-2369-13-166

**Published:** 2012-12-05

**Authors:** Deborah Stuart, Sara Rees, Stephanie K Woodward, Robert Koesters, Kevin A Strait, Donald E Kohan

**Affiliations:** 1 Division of Nephrology, University of Utah Health Sciences Center, 1900 East 30 North, Salt Lake City, UT, 84132, USA; 2 INSERM/Université Pierre et Marie Curie, Paris, France

**Keywords:** Endothelin, Endothelin A receptor, Kidney, Nephron, Sodium, Blood pressure

## Abstract

**Background:**

Endothelin, via endothelin A receptors (ETA), exerts multiple pathologic effects that contribute to disease pathogenesis throughout the body. ETA antagonists ameliorate many experimental diseases and have been extensively utilized in clinical trials. The utility of ETA blockers has been greatly limited, however, by fluid retention, sometimes leading to heart failure or death. To begin to examine this issue, the effect of genetic disruption of ETA in the nephron on blood pressure and salt handling was determined.

**Methods:**

Mice were generated with doxycycline-inducible nephron-specific ETA deletion using Pax8-rtTA and LC-1 transgenes on the background of homozygous loxP-flanked ETA alleles. Arterial pressure, Na metabolism and measures of body fluid volume status (hematocrit and impedance plethysmography) were assessed.

**Results:**

Absence of nephron ETA did not alter arterial pressure whether mice were ingesting a normal or high Na diet. Nephron ETA disruption did not detectably affect 24 hr Na excretion or urine volume regardless of Na intake. However, mice with nephron ETA knockout that were fed a high Na diet had mild fluid retention as evidenced by an increase in body weight and a fall in hematocrit.

**Conclusions:**

Genetic deletion of nephron ETA causes very modest fluid retention that does not alter arterial pressure. Nephron ETA, under normal conditions, likely do not play a major role in regulation of Na excretion or systemic hemodynamics.

## Background

Endothelin-1 (ET-1) has been implicated in the pathogenesis of multiple disorders involving virtually every organ system, including congestive heart failure, pulmonary artery hypertension, atherosclerosis, cancer, autoimmune diseases, chronic kidney disease, and others [1]. The adverse effects of ET-1 are in large part due to interaction with endothelin A receptors (ETA). ETA activation promotes generation of reactive oxygen species, insulin resistance, inflammation, vasoconstriction, cell proliferation, extracellular matrix accumulation, and cellular hypertrophy [2]. In addition, multiple pre-clinical studies have demonstrated that ETA blockade substantially reduces end-organ injury in disease models involving all of the above disorders [2]. Consequently, a large number of clinical trials have been conducted using ET receptor antagonists in heart failure, coronary artery disease, scleroderma, proteinuric renal disease, pulmonary artery hypertension, arterial hypertension, subarachnoid hemorrhage and other disorders [2]. Unfortunately, despite such intense efforts, ET receptor blockers have been approved for only two indications: pulmonary artery hypertension and scleroderma digital ulcers [3].

There are several potential reasons for the failure of these trials, however an almost universal problem has been ET receptor antagonist-induced fluid retention. This fluid retention likely played a significant role in the failure of clinical trials using ET receptor blockers in patients with congestive heart failure. A large Phase III trial using avosentan, a relatively ETA-selective blocker, in patients with diabetic nephropathy was halted due to increased morbidity and mortality associated with drug-induced fluid retention [4]. Atrasentan (a highly ETA-selective blocker) given to a large number of patients with prostate cancer doubled the risk of developing heart failure [5]. While the doses of ET receptor blockers used in these trials were likely substantially higher than necessary, it is evident that ETA blockade-induced fluid retention is a significant problem potentially limiting this class of drug’s clinical utility.

A key question is how ETA blockade causes fluid retention. While ETA antagonism undoubtedly causes some fluid retention in response to vasodilation, this does not explain how patients could become fluid overloaded. A reasonable site for ETA blocker-induced fluid retention is the kidney. Studies in animals indicate that ETA blockade causes renal vasodilation (primarily afferent arteriole) [6], suggesting that a renal hemodynamic effect of these agents does not explain the fluid retention. The ETB receptor has been clearly demonstrated to inhibit thick ascending limb and collecting duct (CD) Na reabsorption, while the role of the nephron ETA receptor in modulating tubule Na and/or water transport remains uncertain [6]. Collecting duct principal cell-specific disruption of ETA did not detectably affect blood pressure (BP) or Na excretion [7], however the role of ETA throughout the nephron in modifying these parameters has never been directly assessed *in vivo*. Consequently, the current study was undertaken, using a recently developed inducible gene targeting model, to determine whether ETA in the nephron can modulate BP or urinary Na excretion.

## Methods

### Animal use assurance

All animal use and welfare adhered to the NIH Guide for the Care and Use of Laboratory Animals following protocol reviews and approval by the Institutional Laboratory Animal Care and Use Committees of the University of Utah Health Sciences Center.

### Generation of inducible nephron-specific ETA knockout mice

**Figure 1:**
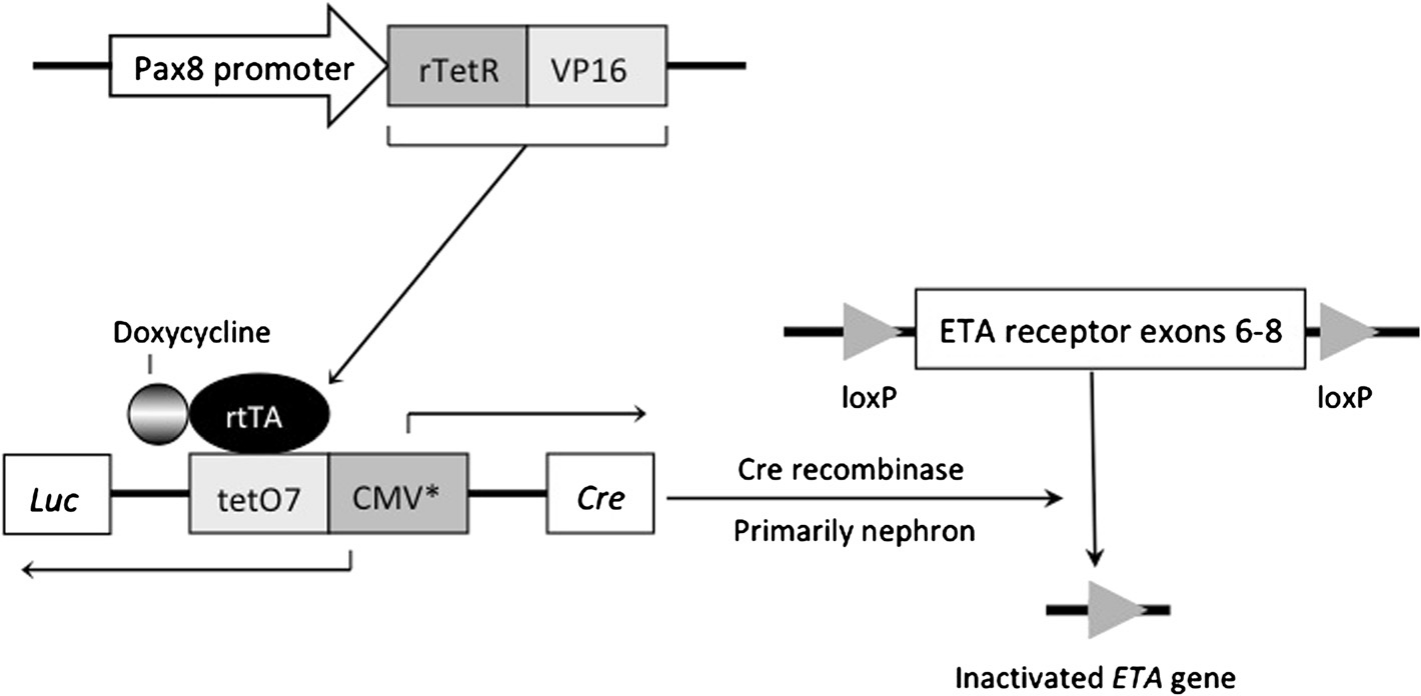
**Schema of conditional KO of the *ENDRA *gene.** The Pax8 promoter drives expression of rtTA which requires doxycycline to activate the bicistronic Cre recombinase and luciferase-expressing transgene. Cre is expressed specifically in the nephron but not in glomeruli. ETA receptor KO mice are homozygous for the floxed *ENDRA* gene and heterozygous for the two transgenes.

Mice capable of inducible nephron-specific ETA knockout (KO) were achieved by breeding mice containing the Pax8-rtTA and LC-1 transgenes with mice containing loxP-flanked (floxed) exons 6–8 of the *EDNRA* gene [7] (Figure 1). The Pax8-rtTA transgene contains 4.3 kb of the *Pax8* gene promoter along with exon 1, intron 1, exon 2 and part of intron 2 driving expression of the reverse tetracycline transactivator [8]. The LC-1 transgene encodes tetracycline-inducible bicistronic Cre recombinase and luciferase [8]. Mice heterozygous for Pax8-rtTA, heterozygous for LC-1, and homozygous for the floxed *EDNRA* gene are termed inducible ETA (iETA) throughout the manuscript. To obtain ETA KO in the nephron, iETA mice were given 2 mg/ml doxycycline (DOX) in 2% sucrose drinking water for 11 days, followed by 4 days off doxycycline (recovery period) before conducting physiologic studies. Equal numbers of male and female mice were used in all studies.

### Genotyping

Tail DNA was PCR amplified and the following primers were used for genotyping: ETA - F 5^′^-cccatgcttagacacaaccatg-3^′^ and R 5^′^-gatgacaaccaagcagaagacag-3- which yield a 364 bp product for the floxed *EDNRA* gene (includes loxP site) and a 324 bp product for the wild type *EDNRA* gene; Pax8-rtTA - F 5^′^-ccatgtctagactggacaaga-3^′^ and R 5^′^-catcaatgtatcttatcatgtctgg -3^′^ which yields a 600 bp product; and LC-1 - F 5^′^-tcgctgcattaccggtcgatgc-3^′^ and R 5^′^-ccatgagtgaacgaacctggtcg-3^′^ which yields a 480 bp product.

### Screening for recombination

DNA from selected organs was PCR amplified to evaluate target organ recombination using primers spanning exons 6–8 in the *EDNRA* gene: F 5^′^-cccatgcttagacacaaccatg-3^′^ and R 5^′^-cgctgttgtatatccagtatcagg-3^′^. Recombination of the *EDNRA* gene yields a 610 bp product; the predicted size of the unrecombined wild type *EDNRA* gene is 1287 bp and, under the PCR conditions utilized, did not yield detectable amounts of product.

DNA from microdissected glomeruli, proximal tubules, thick ascending limbs and cortical collecting ducts was also analyzed for *EDNRA* gene recombination. Kidney slices were incubated with DMEM:F12 containing 2 mg/ml collagenase and 2 mg/ml hyaluronidase for 30 min, then the media replaced with ice-cold HBSS containing 15 mM HEPES and 1% FBS. Tubules were dissected free and individual nephron segments transferred to ice-cold HBSS + 15 mM HEPES; samples were frozen until analysis. DNA was isolated using a microDNA isolation kit (Qiagen, Valencia, CA).

### Expression of mRNA

Total liver, and cortex and inner medulla from homozygous floxed ETA mice without the Pax8-rtTA or LC-1 transgenes (referred to hereafter as “control” mice) and ETA KO (DOX-treated iETA) animals were dissected, and RNA was extracted and reverse transcribed. The resulting cDNA was assayed for relative expression of ETA mRNA in control and ETA KO animals using the Taqman Gene Expression Assay (Applied Biosystems, Carlsbad, CA, ETA probe cat# Mm01243722_m1, GAPDH probe cat# Mm03302249_g1).

RNA from microdissected tubules from control and ETA KO mice was analyzed for ETB mRNA content. Tubules were obtained as described above, RNA isolated and reverse transcribed, and ETB mRNA determined using the Taqman probe cat# Mm00432989_m1, normalizing to GAPDH as described above.

### Immunostaining

Mouse kidneys were perfused with phosphate-buffered saline, then fixed for 1 day in 10% formalin and stored in ethanol. Kidneys were paraffin embedded and 4 μm sections obtained. Sections were deparaffinized on a Benchmark XT (Ventana Medical Systems, Sunnyvale, CA) using EasyPrep (Ventana), and Protease-2 (Ventana) digested for 8 min. Sections were incubated with a 1:100 dilution of rabbit anti-rat ETA (AER-001, Alomone Labs, Jerusalem, Israel) for 2 hr at 37°C, blocked with Sniper (BIocare Medical, Concord, CA) for 4 min, then incubated with a 1:100 dilution of goat anti-rabbit IgG (Sigma, St. Louis, MO) for 32 min. Goat IgG was detected using IView-DAB (Ventana) and sections were counterstained with hematoxylin for 8 min.

### Metabolic cage studies

Control and iETA mice were given free access to water and a normal salt (0.3% Na) diet for 3 days. On the third day, mice were placed in metabolic cages (Hatteras Instruments, Cary, NC) for 24 hr with free access to food and water and urine collected. Tail vein blood (50 μl) was obtained at the end of the 24 hr period. Mice were then fed a high salt (3.2% Na) diet for 7 days with metabolic cage studies performed on the third and seventh days of the high Na diet. Subsequently, both control and iETA mice were given DOX and, after the treatment and recovery period (~2 weeks), the dietary procedures and metabolic cage studies were repeated.

Urine Na was determined on an EasyVet Analyzer (Medica, Bedford, MA). Urine and blood creatinine was determined using the QuantiChrom Creatinine Assay Kit (BioAssays Systems, Hayward, CA).

### Blood pressure monitoring

Blood pressure was monitored in control and iETA mice by radiotelemetry (TA11-PAC10, Data Sciences International, St. Paul, MN) with catheters inserted into the right carotid artery. The mice were allowed to recover for 1 week after surgery. BP and heart rate and were monitored continuously. Mice were fed normal and high Na diets as described under the metabolic studies, and these were repeated after DOX administration. The BP studies were conducted separately from the metabolic cage studies, using different mice, in order to minimize handling of animals during hemodynamic recordings.

### Fluid retention analysis

The iETA mice were fed a normal Na diet for 7 days, followed by a high Na diet for 7 days. This time schedule was used to insure maximal stability of mice, i.e., giving them a full week on each diet to adjust their body fluid volume. Mice were then treated with DOX or vehicle after which the normal and high Na diets were repeated. On the 7th day of each diet, body weight was determined. A 20 μl blood sample was obtained for determination of hematocrit. Body compartment fluid volume was then determined by impedance plethysmography as previously described [9]. Briefly, mice were anesthetized and measured for length and width. Four needles were inserted under the skin at the base of the tail, the intercept between the front of the ears and the longitudinal midline, and 0.5 cm from these sites toward the tip of the tail and the nose, respectively. Leads from the needles were attached to the ImpediVet BIS1 system (ImpediMed, San Diego, CA) that analyzes whole body bioimpedance data to determine total body water (TBW), extracellular fluid volume (ECF) and intracellular fluid volume. A resistance coefficient equal to 10% of that for rats was used for all mice studies.

### Statistical analysis

Data are presented as mean percent of control or as absolute values, either one ± SE. Data were compared using one- or two-way ANOVA as indicated. The criterion for significance was p < 0.05.

## Results

### Confirmation of inducible nephron-specific *EDNRA* gene knockout

**Figure 2:**
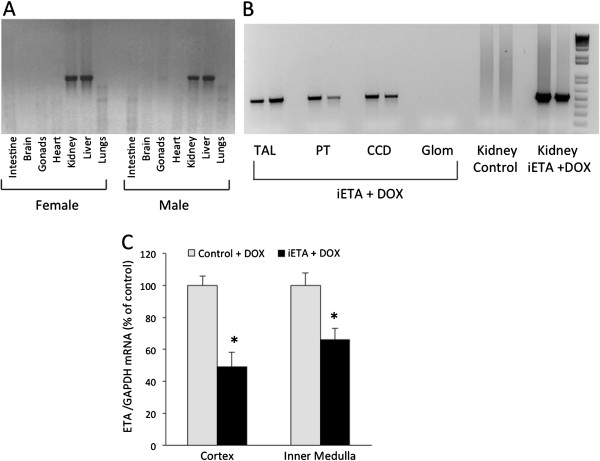
**Specificity and sites of ETA receptor KO.** Panel **A** shows a representative blot (N = 6 each gender) of PCR for *ENDRA* gene recombination, demonstrating Cre activity in liver and kidney. Panel **B** shows a representative blot (N = 8) of PCR for *ENDRA* gene recombination in microdissected thick ascending limb (TAL), proximal tubule (PT), cortical collecting duct (CCD) and glomeruli from Pax8-rtTA/LC1/floxed ETA (iETA) mice treated with doxycycline (DOX) (separate mice shown in each lane). Negative (homozygous floxed ETA (control)) and positive (iETA + DOX) PCR of whole kidney DNA are shown. Panel **C** shows real-time PCR of ETA mRNA (normalized to GAPDH mRNA) in cortex and inner medulla from control + DOX and iETA + DOX mice (N = 8 each group). *p < 0.05 vs. control + DOX.

Mice heterozygous for Pax8-rtTA, heterozygous for LC-1, and homozygous for the floxed *EDNRA* gene (inducible ETA KO (iETA)) manifested no developmental abnormalities, lived up to at least 1 year of age, and had no apparent renal or hepatic histologic abnormalities up to at least 1 year of age. Please note that the terms “iETA” and “ETA KO” are not interchangeable: iETA mice become ETA KO when given DOX. Treatment of iETA mice with DOX caused *EDNRA* gene recombination in liver and kidney, but not in other organs (Figure 2A). This finding is consistent with the original description of Pax8-rtTA transgene activity, with relatively small periportal hepatocyte activity as compared to extensive renal activity [8]. DOX treatment caused *EDNRA* gene recombination in proximal tubules, thick ascending limbs, and cortical collecting ducts, but not in glomeruli (Figure 2B). Thus, as expected, the DOX-treated iETA mice had primarily nephron-specific KO of the *EDNRA* gene. The iETA mice not exposed to DOX manifested no evidence of genomic recombination in the kidney or elsewhere (data not shown).

**Figure 3:**
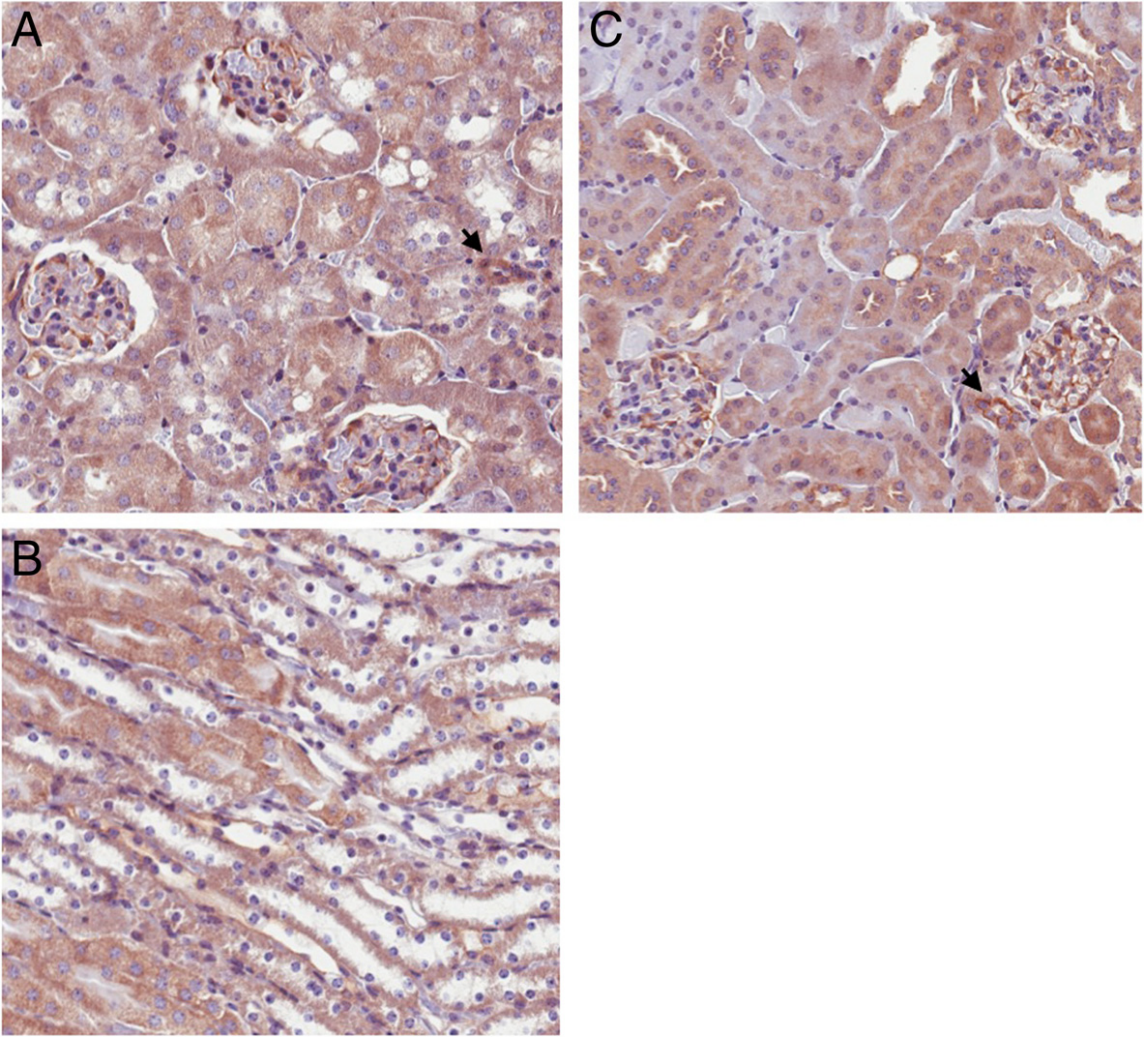
**Representative photomicrographs of kidneys from Pax8-rtTA/LC1/floxed ETA (iETA) mice before and after doxycycline (DOX) treatment.** Panel **A** shows cortex and Panel **B** shows medulla prior to DOX treatment; Panel **C** shows cortex after DOX treatment. DOX-treated iETA medullary staining is not shown since ETA immunostaining was not apparent in untreated iETA mice. Arrows indicate blood vessels. Staining was performed on 6 different mice of each genotype. Images are 200X.

The iETA KO mice treated with DOX had a 50% reduction in ETA mRNA in renal cortex and about a 40% decrease in ETA mRNA in renal inner medulla as compared to controls given DOX (mice homozygous for the floxed *EDNRA* gene, but not containing the Pax8-rtTA or LC-1 transgenes) (Figure 2C). Total liver ETA mRNA was not reduced in DOX-treated iETA mice (data not shown). Since ETA is most intensely expressed in the vasculature and glomeruli, such incomplete reduction of renal ETA mRNA levels in ETA KO mice is anticipated. To further demonstrate the specificity of renal ETA knockout, Figure 3 shows representative images of renal ETA expression as detected by immunohistochemistry in iETA mice before and after DOX treatment. In untreated iETA mice, cortical ETA staining was evident primarily in glomeruli and blood vessels (Figure 3A), while medullary ETA staining was not detectable (Figure 3B). DOX-treated iETA mice showed no reduction in the intensity of glomerular or vascular ETA staining (Figure 3C), supporting the findings that these regions were not affected in ETA KO animals. In addition, these findings indicate that nephron ETA is in relatively low abundance compared to glomeruli and vascular tissue, and not readily detectable by immunostaining.

### Effect of nephron ETA KO on blood pressure and urinary Na and water excretion

**Figure 4:**
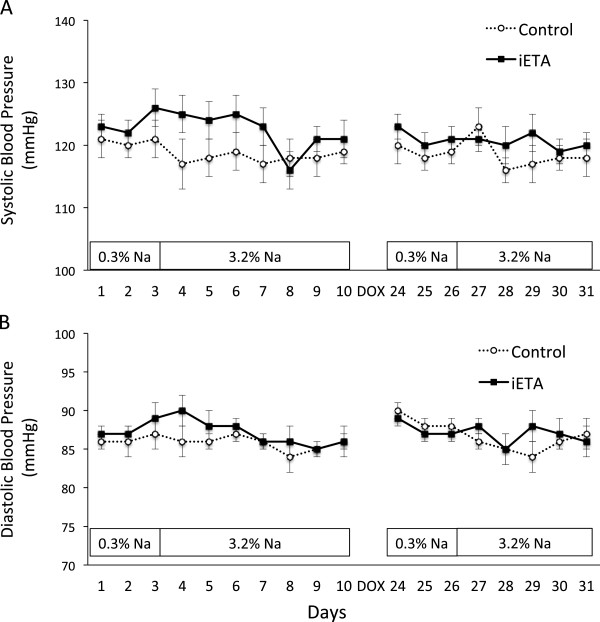
**Systolic (Panel A) and diastolic (Panel B) BP in control and Pax8-rtTA/LC1/floxed ETA (iETA) mice before and after doxycycline (DOX) treatment.** Animals were fed a normal (0.3%) Na diet for 3 days followed by a high (3.2%) Na diet for 5–7 days. DOX was given for 11 days, then BP recordings continued 5 days after discontinuation of DOX. BP was obtained by continuous radiotelemetry. Daily BP values are shown as the average of the every 10-minute recordings throughout that 24 hr period. N = 12 each data point.

BP was monitored continuously on normal and high Na diets in control (homozygous for floxed ETA) and iETA mice before and after DOX administration. As shown in Figure 4, systolic (Panel A) and diastolic (Panel B) BP were not different between control and iETA mice before administration of DOX regardless of the amount of Na intake; DOX administration did not result in different systolic or diastolic BP between the two groups regardless of the level of Na intake nor did it change BP as compared to pre-DOX values; there was no difference between genders in terms of the BP response to DOX. Note that BP values shown for each day are an average of all values obtained over the course of that day (values are recorded every 10 minutes). The expected diurnal variation of BP occurred in both groups before and after DOX administration, and the pattern of this was similar between groups (data not shown).

**Figure 5:**
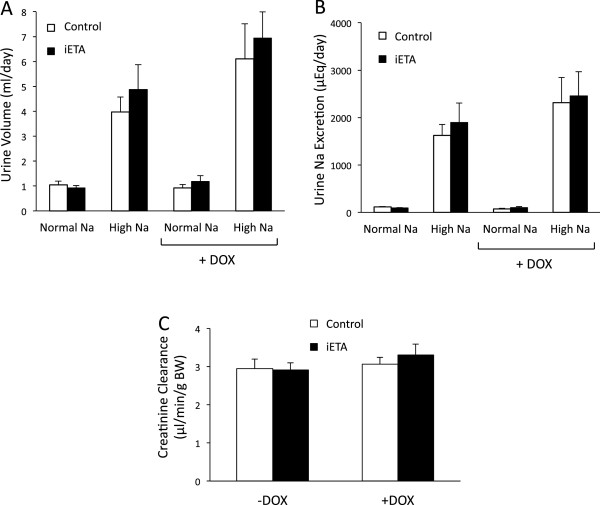
**Urine volume (Panel A), urinary Na excretion (Panel B) and creatinine clearance (Panel C) in control and Pax8-rtTA/LC1/floxed ETA (iETA) mice before and after doxycycline (DOX) treatment.** Animals were fed a normal (0.3%) Na diet for 3 days followed by a high (3.2%) Na diet for 7 days. Urine (24 hr) was obtained on the third day of starting normal or high Na diets. Note that within each panel, open or closed bars represent the same mice, i.e., mice were sequentially given a normal and high Na diet, treated with DOX, and the diets repeated. N = 8 each data point.

Urine volume (Figure 5A) and urinary Na excretion (UNaV) (Figure 5B) were similar between control and iETA mice on a normal or high Na intake, regardless of gender. There were also no differences in urine volume or UNaV between the two groups of mice after DOX administration regardless of Na intake. The change in urine volume or UNaV (comparing before and after DOX administration) was also not different in the two groups of mice (data not shown). Urine volume and UNaV were also determined on the seventh day of normal or high Na diets in both groups of mice before and after DOX administration; as for the day 3 values, there were no differences in Na excretion observed between groups regardless of Na intake (data not shown). Water and Na intake were not different when comparing before vs. after DOX, nor were they different comparing control vs. iETA mice (data not shown). Finally, creatinine clearance was not different between control and iETA mice before or after DOX treatment (Figure 5C). It is recognized that creatinine clearance is not a highly accurate measure of glomerular filtration rate in rodents, so this was only used as a rough indicator of renal function.

### Fluid retention analysis in iETA mice

**Figure 6:**
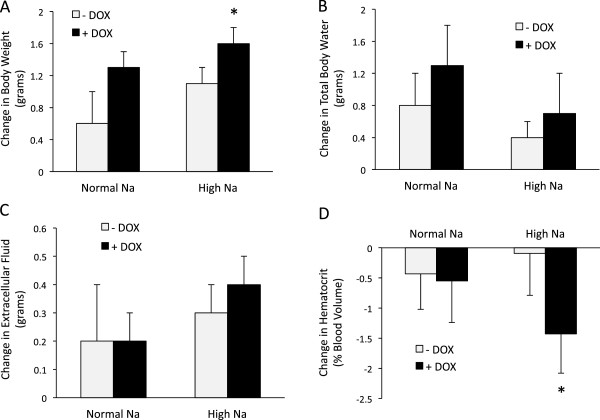
**Change in body volume status in Pax8-rtTA/LC1/floxed ETA (iETA) mice comparing before and after vehicle or doxycycline (DOX) treatment.** Animals were fed a normal (0.3%) Na diet for 7 days followed by a high (3.2%) Na diet for 7 days, then treated with DOX, and the diets repeated; measurements were obtained on the 7th day of each diet. Panel **A** shows change in body weight, Panels **B** and **C** show changes in total body water and extracellular fluid volume, respectively, as assessed by impedance plethysmography, and Panel **D **shows the change in hematocrit. N = 8 each data point. *p < 0.05 vs. –DOX, same diet.

The above studies found no difference in 24 hr urinary water or Na excretion between groups, however small differences in salt or water excretion that might exert a cumulative effect on body fluid volume might not be detected. Consequently, additional studies were conducted in order to detect small changes in body fluid volume. Figure 6A shows the change in body weight in iETA mice where the change being measured is the body weight after DOX (+DOX) or vehicle (−DOX) treatment compared to the body weight pre-treatment. Starting weights of mice not receiving DOX were not different from starting weights of mice later treated with DOX (24.2 ± 0.8 g and 24.9 ± 0.8 g, respectively). Comparing body weight on a normal Na diet, iETA animals receiving DOX tended to have a greater increase in body weight than iETA animals receiving vehicle, i.e., nephron ETA KO tended to increase body weight on a normal Na diet. The percent change in body weight over the 1 week of the normal Na diet also tended to be greater in DOX-treated mice, but did not achieve statistical significance (weight increase of 2.7 ± 1.8% in -DOX and 4.8 ± 1.4% in + DOX). Notably, ETA KO caused a rise in body weight that was significantly greater than that seen in vehicle-treated iETA mice when animals were fed a high Na diet. Similarly, the percent change in body weight was greater in DOX-treated than non-treated iETA mice on a high Na diet (weight increase of 4.1 ± 0.8% in –DOX and 7.0 ± 0.6% in + DOX). Total body water tended to increase more in DOX-treated iETA mice as compared to vehicle-treated animals regardless of the Na intake, however this did not achieve statistical significance (Figure 6B). No significant effect of DOX treatment on extracellular fluid volume was detected (Figure 6C). Hematocrit fell to a greater extent in DOX-treated mice on a high Na diet as compared to vehicle-treated mice (Figure 6D), consistent with enhanced hemodilution due to fluid retention. DOX given to control (homozygous floxed ETA) mice did not affect any of these parameters, ruling out an effect of DOX itself (data not shown). Finally, there were no differences between genders in terms of the above responses to DOX. Taken together, these data suggest that nephron ETA KO causes modest fluid retention that is evident when Na intake is high.

### Effect of nephron ETA KO on nephron ETB mRNA

ETB mRNA content was determined in proximal tubules (PT), thick ascending limbs (TAL) and cortical collecting ducts (CCD) from iETA mice treated with vehicle or DOX. ETB mRNA levels were detected in the following order: CCD > TAL > PT. The deltaCT, defined as cycle number at which ETB is first detected minus the cycle number at which GAPDH is first detected (so the lower the deltaCT, the more ETB mRNA is present), was 11.22 ± 0.27 for PT, 9.44 ± 0.32 for TAL, and 7.67 ± 0.19 for CCD, N = 12 each nephron segment. Nephron ETA KO did not significantly affect ETB mRNA in CCD (99 ± 8% of control), TAL (76 ± 19% of control) or PT (79 ± 13% of control) wherein 6 different tubules from 3 mice in each group were analyzed.

## Discussion

The first major finding in the current study is that nephron ETA can modulate volume homeostasis, albeit their normal physiological role appears to be very modest. An increase in body weight and fall in hematocrit indicate fluid retention after nephron ETA KO, however such fluid retention was too small to permit detectable changes in body fluid volume compartments or in urinary Na excretion. The latter measurements (body fluid compartments and urinary Na excretion) have rather high intrinsic variability, making detection of significant changes challenging. Nonetheless, at least on a high Na diet, total body water and extracellular fluid compartments tended to be increased in nephron ETA KO animals.

Nephron ETA KO causes mild fluid retention, suggesting that nephron ETA exerts a natriuretic effect. Previous studies have clearly implicated the ETB receptor in the proximal tubule, thick ascending limb, cortical collecting duct (CD), and inner medullary CD as mediating ET-1 inhibition of Na and/or water reabsorption [6]. In contrast, CD-specific ETA KO mice do not manifest alterations in BP or UNaV (although subtle changes in volume regulation could have been missed) [7]. Similarly, ETA blockers do not affect ET-1 inhibition of epithelial Na channel (ENaC) activity in isolated cortical CD [10] nor is ENaC activity in this nephron segment altered in CD ETA KO mice [11]. However, there is data to suggest that renal ETA can exert a natriuretic effect. Mice with CD ETB KO are modestly hypertensive and retain Na [12], while combined CD ETA and ETB KO mice are significantly more hypertensive and retain more Na than mice with CD ETB KO alone [13]. In addition, intra-renal medullary administration of ET-1 to rats deficient in ETB causes a natriuresis and diuresis and this effect is prevented by an ETA antagonist [14]. Taken together, these studies suggest that ETB is the primarily mediator of ET-1 inhibition of nephron Na and water transport, but that ETA can also exert a natriuretic effect.

The mechanism(s) by which nephron ETA exerts a natriuretic effect and the precise sites in the nephron at which this occurs remain unknown. An intriguing possibility is that ETA might affect ETB activity as suggested by the finding that combined CD ETA and ETB KO mice retain more Na than mice with KO of either receptor alone [13]. ETA/B heterodimerization has been described *in vitro* and such heterodimerization can affect ET receptor trafficking and potentially signaling [15,16]. While the goal of the current study was to determine whether nephron ETA could affect renal Na handling, clearly additional studies are necessary to dissect out the mechanism(s) responsible for this effect.

It remains to be determined how ETA antagonists cause fluid retention, however the current studies suggest that blockade of nephron ETA may pay a role. Given that the ETA KO phenotype is very mild, it may be that blockade of ETA outside of the nephron may also be involved in ETA antagonist-induced fluid retention. Despite the disappointing results with earlier clinical trials, ETA blockers still hold promise for the treatment of a variety of diseases. As mentioned earlier, multiple pre-clinical and early phase clinical trials suggest that ETA blockers may be of therapeutic benefit in a wide variety of disorders [2]. A recent phase IIA trial in patients with diabetic nephropathy using atrasentan in doses that caused only modest fluid retention showed significant reductions in albuminuria over an 8-week period [17]; further trials are planned to determine whether ETA blockade slows progression of diabetic kidney disease. Although the primary endpoint of reduction in office BP was not met, darusentan significantly reduced ambulatory BP in patients with resistant hypertension as compared to guanfacine [18]. Trials are actively recruiting for patients to study the effect of ETA antagonists in myocardial infarction [19], cancer [20], and other disorders. Thus, determination of the sites and mechanisms responsible for ETA antagonist-induced fluid retention remains an important question.

## Conclusions

Using an inducible Cre-based strategy, we found that disruption of ETA receptors in the nephron causes mild Na retention with no detectable effect on BP. These findings indicate that ETA receptors in the nephron, per se, are potentially involved, at least partially, in ETA antagonist-induced fluid retention. Key next steps involve determination of the sites and mechanism(s) of nephron ETA regulated Na transport, as well as how ETA blockers exert their fluid retaining effects.

## Competing interests

D.E.K. has served as a consultant for Abbott Laboratories and Gilead Sciences, Inc.

## Authors' contributions

DS performed the arterial pressure and Na balance studies, SR performed the RNA analysis, SW performed the DNA analysis, RK developed and provided the Pax8-rtTA and LC-1 mice, KAS supervised the breeding and analysis of mouse genotypes, and DEK designed the study, analyzed the data, and supervised the project. All authors read and approved the final manuscript.

## Pre-publication history

The pre-publication history for this paper can be accessed here:

http://www.biomedcentral.com/1471-2369/13/166/prepub
